# Considerations Using Harmaline for a Primate Model of Tremor

**DOI:** 10.5334/tohm.634

**Published:** 2021-09-13

**Authors:** Edward M. Bello, Madeline Blumenfeld, Joan Dao, Jordan D. S. Krieg, Lucius K. Wilmerding, Matthew D. Johnson

**Affiliations:** 1 Biomedical Engineering Department, University of Minnesota, US; 2 Institute for Translational Neuroscience, University of Minnesota, US

**Keywords:** tremor, harmaline, preclinical model, non-human primate, accelerometer

## Abstract

**Background:**

While harmaline has been used as a pharmacological model of essential tremor (ET) in rodents and pigs, less is known about the effects of this pharmacological treatment in awake-behaving non-human primates. In this study, we investigated the time-course, amplitude, frequency, and consistency of harmaline tremor in primates.

**Methods:**

Three rhesus macaques were administered doses of harmaline ranging from 2–12 mg/kg (i.m.), and tremorous movements were quantified with accelerometers. One subject was also trained to perform a self-paced cued reaching task, with task engagement assessed under harmaline doses ranging from 2–8 mg/kg (i.m.).

**Results:**

Whole-body tremors manifested within 30 minutes of threshold-dose administration, and peak oscillatory frequency ranged between 10–14 Hz. However, large differences in tremor intensity and intermittency were observed across individual subjects under similar dosing levels. Additionally, engagement with the reaching task was dependent on harmaline dose, with performance mostly unaffected at 2 mg/kg and with little task-engagement at 8 mg/kg.

**Discussion:**

We provide a detailed assessment of factors that may underlie the heterogeneous responses to harmaline, and lay out important caveats towards the applicability of the behaving harmaline-tremoring non-human primate as a preclinical model for ET.

**Highlights:**

The harmaline-primate is revisited for its potential as a preclinical model of tremor. Spontaneous tremor was heterogenous in amplitude across subjects despite similar harmaline doses, action tremors were not consistently observed, and performance on a behavioral task degraded with higher dosages.

## Introduction

Non-human primate models continue to advance basic research in and novel treatments for movement disorders, with examples including the MPTP model of Parkinson’s disease [1], the repetitive strain model of focal hand dystonia [2], and the transgenic model of Huntington’s disease [3]. Non-human primate models of Essential Tremor (ET), on the other hand, have not yet been thoroughly developed or investigated. Essential Tremor (ET) is a human movement disorder characterized by involuntary oscillatory tremors with a postural component [4], kinetic/intention component [5], and/or active task-specific tremor during activities such as writing, pouring, and utensil use, with tremors typically localized bilaterally to the upper limbs [4].

Animal models of action-tremor have been achieved through genetic alterations [6], CNS lesions [7,8], and pharmacological administrations [9]. Pharmacological agents, in particular, have been used to mimic the phenomenology of action tremors, though with some limitations. Cholinomimetics such as oxotremorine and carbachol have been administered in mammals in order to cause generalized tremors, though the observed tremor frequencies generally exceed the range observed in human ET [10,11]. In addition, the induced tremors are not reduced with doses of ethanol, as ET-tremors are known to be [12]. Systemic administration of MPTP, a toxin targeting dopaminergic cells in the substantia nigra pars compacta, can induce a postural tremor in primates [13,14,15], though expression is inconsistent across individuals and within non-primate mammals [16,17]. The most commonly used pharmacological approach in animal models of tremor is the systemic administration of harmaline.

Harmaline, a beta carboline alkaloid, is generally considered to cause action tremors [18], with an oscillatory frequency occurring in the range of 11–14 Hz in mice [19], 8–15 Hz in rats [20,21,22], 8–12 Hz in cats [23], and 10–16 Hz in pigs [24]. Human ET has been reported in the 4–12 Hz range [4]. Similarly to ET tremors, harmaline tremors can be attenuated by ethanol [12] and by pharmacological agents prescribed for ET [25]. A wide range of harmaline doses have been used to induce tremors across mammals (0.5–100 mg/kg) [11], with differing tremor onset times and intensities depending on the species, dose, and the route of administration (i.v., i.m., i.p., s.c.). Cats typically respond with visible tremors to a harmaline dose of 5 mg/kg (i.m.) [11], while effective doses have been reported from 9 to 50 mg/kg in rats, with various routes of administration [20,21], and 2–6 mg/kg (i.v.) in pigs [24]. The effects of harmaline and dosages necessary to generate tremor in non-human primates have not been thoroughly investigated, with only a few studies having been performed to observe the effects of harmaline with CNS lesioning [26,27,28,29,30] and to probe the oculomotor system [31,32].

In attempting to phenomenologically mimic human ET tremors, the use of a harmaline-primate model of tremor has potential. The close correspondence in musculoskeletal anatomy between humans and non-human primates can facilitate movement quantification, comparisons of tremor-kinematics across similar scales and joint-configurations to humans that cannot be replicated in other non-primate models. Additionally, human brain structures in the motor control network have a tighter correspondence with primate brain anatomy than with other non-primate organisms. Furthermore, in focusing on the pathophysiological changes induced by harmaline, the ability to both invasively record neural activity and invasively modulate neural activity (e.g. deep brain stimulation) is more readily accomplished in large animal models that offer greater brain volumes to accommodate experimental therapeutic devices. Finally, primates have a unique advantage in the potential to adequately model the task-related action-tremor signs observed in most human ET patients during goal-oriented behavior, which is far more difficult to translate to other animal models of tremor [21]. A behaving harmaline-primate tremor model would allow for careful dissection of the motor task-dependent tremor phenotypes possible under the influence of harmaline.

We conducted a study to characterize the tremor onset and frequency characteristics of the awake rhesus macaque under various doses of intramuscular harmaline administration, and explored its potential as a behaving (i.e. motor task-performing) preclinical tremor model for human ET. We report here on the tremor-characteristics induced by harmaline dosing across 3 subjects, and further report the results of dose-dependent effects of behavioral performance in a self-paced cued reaching task.

## Methods

### Animals

**Figure 1:**
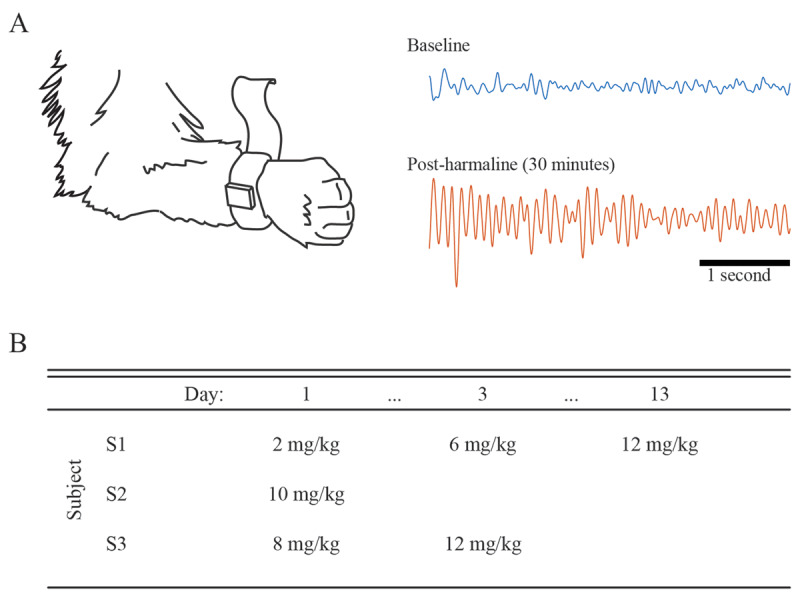
Dosing for tremor responses among subjects. **A.** Acquisition of wrist-mounted accelerometry. Left: Subjects had devices for measuring triaxial accelerometry mounted securely to the wrist using vet wrap. Right: Example data recorded from one axis of an accelerometer during Subject 1’s 10 mg/kg harmaline exposure session (time series are shown on the same amplitude scale, and have been bandpass-filtered between 1 and 30 Hz). **B.** Dosage of harmaline delivered intramuscularly (mg/kg) to a subject during a given harmaline dosing session, arranged in columns according to the time elapsed (days) since the subject’s first harmaline administration of the study (“day 1”).

Three adult rhesus monkeys (*Macaca mulatta; S1: female, 4.6 kg, 14 yr old; S2: female, 6.5 kg, 16 yr old; S3: female, 8.8 kg, 16 yr old)* were used in this study. All procedures were approved by the *Institutional Animal Care and Use Committee* of the University of Minnesota and complied with the United States Public Health Service policy on the humane care and use of laboratory animals. Before initiating this study, S1 had no prior surgeries or equipment present. S2 and S3 had been chronically implanted with cranial chambers and unilateral (S2) or bilateral (S3) thalamic deep brain stimulation leads as part of previous studies [33,34]. Animals were acclimated to sitting in a primate chair and to wearing wrist-mounted accelerometers on one forelimb with full range of motion for the duration of recording sessions (***Figure 1A***). The primates sat in their chairs in an alert state, with occasional changes in seated posture and limb positions, but otherwise did not display much deliberate, goal-oriented movement.

### Harmaline administration

**Figure 2:**
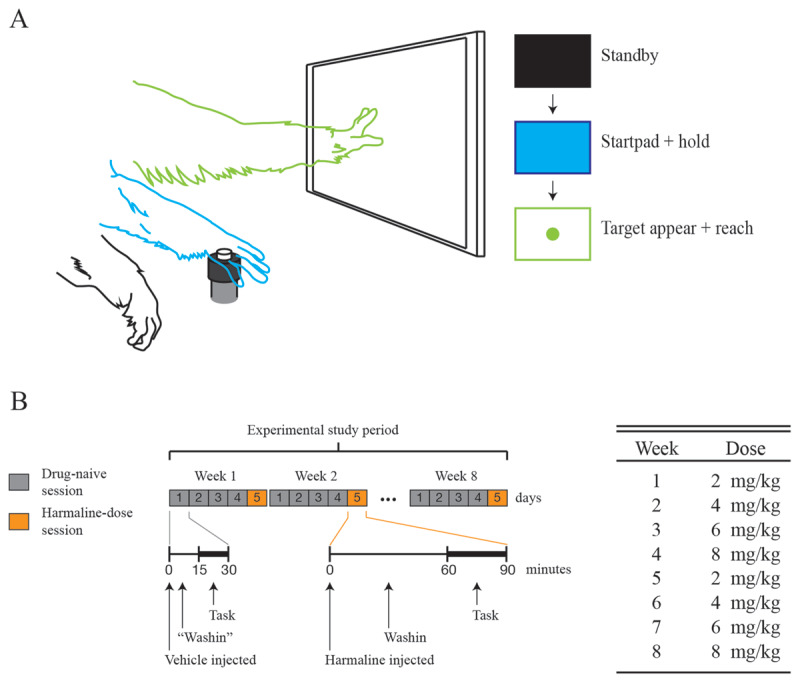
Self-paced cued reaching task performance under harmaline dosing conditions in S3. **A.** Representation of the subject’s engagement with the startpad and touchscreen elements of the self-paced cued reaching task. The illustrated colored limb outlines depict the position of the upper limb according to the corresponding stage of each reach-trial. In Standby mode, the subject was not engaged with the task, and the monitor displayed a solid-black screen. Once the task was initiated and the subject was in the hold-phase, the monitor displayed a solid-blue screen until the variable hold period was completed. Finally, the circular target appeared in the center of the screen and the subject could reach and touch the target for a liquid reward. **B.** Overview of the structure and time course of the self-paced cued reaching task study, consisting of one experimental session per day, 5 consecutive days per week, for 8 weeks. Task duration and preceding washin waiting time after injection is depicted, as well as the correspondence between each week in the study and the harmaline dose delivered during that week.

Subjects were treated with a range of systemic harmaline doses to investigate harmaline-induced tremor dosimetry (***Figure 1B***). Harmaline hydrochloride salt (Sigma Aldrich, St. Louis MI) was diluted in sterile water, prepared in sterile conditions on the day of exposure, and delivered by intramuscular injection into the lateral aspect of the quadriceps muscle group. Doses (mg/kg) were prepared immediately prior to recording sessions and protected from direct light. Total injection volumes varied between 0.4 and 1.75 mL of solution, distributed at times across multiple syringes and delivered across both limbs so that individual injection volumes did not exceed 0.5 mL. An additional exposure study was performed in one subject approximately 18 months later to assess the effect of harmaline dose on task-performance where injection volumes were controlled to ~0.4 mL for each dose (***Figure 2B***).

### Accelerometry acquisition

During harmaline treatment sessions, tremor-specific and general behavioral observations were noted while wrist-mounted triaxial accelerometry was collected at irregular intervals. All accelerometry recordings ranged between 1–2 minutes in duration, depending on the recording system used. For S1 and S2, data acquisition was based on finite sample counts. In the case of S3, data acquisition was continuously streamed, and recordings longer than 2 minutes were segmented into smaller data windows as described below. The hardware and software used for data acquisition included: (*S1*) accelerometry measured with a G-link wireless accelerometer node and Micro TxRx wireless base station, saved to a PC using Agile-Link software (Microstrain, Williston VT), and acquired at 617 samples/second; (*S2*) the same accelerometer attached to the forelimb contralateral to the aforementioned lead implantation site, and wireless station with acquisition managed by a NI USB-6211 multifunction DAQ (National Instruments, Austin TX) connected to the wireless station’s analog output and acquired at 500 samples/second; (*S3*) triaxial accelerometer (model: 356B21; PCB Piezotronics Inc., Depew NY) connected to ADC channels of a TDT System 3 hardware (RZ2 BioAmp Processor) and software (Synapse) setup, acquired at 1017.3 samples/second (Tucker Davis Technologies, Alachua FL).

### Data analysis

Acquisition resulted in recordings saved in disparate digital formats, therefore all data was converted into a common format laid out by the Neurodata Without Borders project [35] to facilitate the use of a common analysis pipeline for subject data. All further analysis was performed using custom-written scripts in Matlab (version 2018a, Mathworks, Natick MA).

#### Data processing and movement artifact rejection

Triaxial accelerometer data was detrended by subtracting the best linear fit (Matlab detrend function) and high-pass filtered with a 6th order Butterworth filter at a 1 Hz cutoff frequency. A spectrogram was obtained for each of the three channels of triaxial accelerometry using the short-time Fourier transform with 1-second Hamming windows and no overlap. The three spectrograms were summed so that time-frequency contributions from all three orthogonal axes of acceleration were accumulated within one combined spectrogram. Since brief episodes of sudden jerky movements caused a broad spectral leakage of high-power components in spectrograms, such artifactual periods were excluded from analysis. For all 1-second windows of spectrogram data across all recordings, summed spectral power of each window was obtained by summing all spectrogram data along the frequency dimension. Any given 1-second window of summed spectral power outside 3 median absolute deviations (relative to the median of all data in the session) was excluded from further analysis. Remaining non-artifactual periods of data were concatenated in time, after being individually detrended to mitigate temporal discontinuity in the data.

#### Tremor-band motion power ratio

For the above-mentioned 1-second windows of spectrogram data derived from accelerometry, bands of frequency indicative of strong oscillatory activity were designated as “tremor bands” of interest (see Results for specific tremor-bands). In order to observe the evolution of tremor band power over time while accounting for inter-subject differences, we calculated a motion power ratio (MPR) measure similar to previous reports [24,25]. In our application, MPR was defined as the power in a tremor-band of interest divided by the power in non-tremor frequencies (i.e. tremor-power / [17,18,19,20,21 Hz]-power).

#### Power spectral density estimation

The power spectral density (PSD) of each processed data segment was estimated using Welch’s method (Matlab pwelch function; 4 second windows). The frequency-resolution of PSDs for each subject were as follows: S1, 0.1506 Hz; S2, 0.2441 Hz; S3, 0.2484 Hz. Peak frequencies from each data segment’s estimated PSD were tracked.

### Self-paced cued reaching task and dosing regimen

Following a period of ~18 months after the aforementioned harmaline exposure study, Subject S3 participated in an additional study which consisted of performing a self-paced cued reaching task across a range of four systemic harmaline doses of 2, 4, 6, and 8 mg/kg (***Figure 2A***). The reaching task entailed touching a target on a touchscreen upon its appearance with one free hand. The subject’s total number of attempted reaches within the allotted session time were tracked as a measure of task-engagement. The number of reaches initiated prematurely before the appearance of the touchscreen target were tracked as a measure of impulsivity. The task study schedule consisted of one task session per day, 5 consecutive days per week, for 8 weeks (***Figure 2B***). Daily sessions were either harmaline-dose sessions (1 day of the week), which began with injection of harmaline solution in water, or drug-naive sessions (4 days of the week), which began with injection of an equivalent volume of vehicle (saline). Doses were prepared and administered intramuscularly. On harmaline-dose days, the task did not commence until a 60-minute wash-in period post harmaline-injection had elapsed, to provide enough time for adequate uptake of harmaline at all doses. On drug-naive days, 15 minutes of wash-in post saline injection were allowed before commencing the task, as a modicum of consistency with harmaline-dose days. On harmaline-dose days, the subject was given 30 minutes to perform all desired task trials for a liquid reward (water). On drug-naive days, the subject was given 15 minutes to perform task trials, though the subject always reached liquid reward satiety before 15 minutes had elapsed. Since the first exposure to 8 mg/kg of harmaline resulted in very little engagement with the task in S3, higher doses were not investigated.

### Statistics

*Drifting trend in time for detected PSD frequency peaks*: the time-drifting trend in the frequency of detected peaks in PSD estimates was fit with a first-order linear equation using Matlab’s csfit toolbox, limited to data recorded after 15 minutes post-injection, after outlier-exclusion. *Normalized MPR over time*: significant changes in harmaline-tremor MPR from baseline were tested as follows. Any data recorded before harmaline-injection or within 15 minutes of injection was considered baseline activity. The distribution of MPR values for each segment of recorded data was tested for significant difference from median baseline MPR using the Wilcoxon signed-rank test. The false discovery rate due to testing multiple data segments in each recording session was controlled using the Benjamini-Hochberg procedure (FDR = 0.0001, relatively conservative).

## Results

### General harmaline response characteristics in seated primates

The dosages delivered to each subject and the relative time intervals (days) between exposures are depicted in ***Figure 1B***. Two of the three subjects (S1, S2) demonstrated robust harmaline-tremors at the 10–12 mg/kg dosages. When given the 2 mg/kg and 6 mg/kg doses, S1 did not present with appreciable oscillatory tremor-like signs, either immediately perceivable to observers or later in offline accelerometry analysis. Systemic harmaline doses of 12 mg/kg and 10 mg/kg resulted in consistent oscillatory whole-body tremors in S1 and S2, respectively. S3 did not exhibit a consistent tremor at either of the initial doses tried (8 and 12 mg/kg), though infrequent low-amplitude, transient bouts of tremulous activity were observed. All further analysis focused on the session with maximum administered dose for each subject (***Figure 1B***).

For all three subjects, harmaline doses between 8–12 mg/kg were noted to cause mild akathisia (inability to sit still) and piloerection prior to the manifestation of harmaline tremors, though these effects wore off within an hour. Nystagmus was observed through the duration of recordings at these harmaline doses, with S3 expressing an occasional brief, transient esophoria in the eyes. At these doses, subjects occasionally exhibited signs of nausea during recordings and difficulties with balance in their home enclosure. Both immediate and post-study monitoring of subjects did not indicate any muscle weakness or loss of function in the harmaline-administered musculature.

### Heterogeneous frequency characteristics in tremor-responses to similar doses of harmaline across individual awake primates

**Figure 3:**
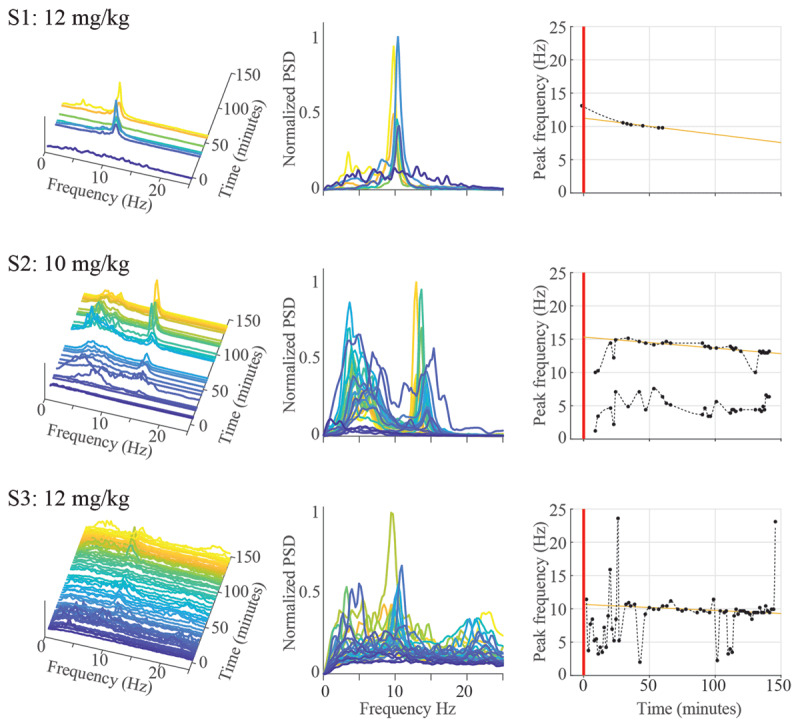
Spectral content of the forelimb accelerometry recordings following harmaline injection in each subject, at tremorgenic doses. These data are presented in terms of (left) power spectral densities color coded and arranged in time since harmaline injection, (middle) power spectral densities with the same color coding stacked to emphasize spectral peaks, and (right) peak frequency in the 10–14 Hz range plotted over time. Solid lines in the rightmost figures indicate the linear regression fit for peak frequency over time.

Accelerometry data was further analyzed for the highest harmaline dosages tested in each subject: S1: 12 mg/kg; S2: 10 mg/kg; S3: 12 mg/kg. Power spectral density estimates (PSDs) for S1 exhibited a narrow oscillatory peak centered at ~10 Hz, while S2’s manifested a similar peak at ~14 Hz as well as a more diffuse spectral power peak centered at ~6 Hz (***Figure 3***). Following the initial appearance of tremor, the power of these oscillatory features increased, and the bandwidth of these features narrowed over time. Over the period of accelerometry recorded, tremor power fluctuated considerably, but in all subjects tremors were generally seen to gradually decrease in power and consistency, subsiding totally within at most 5 hours post-injection time. For S3, PSDs exhibited an intermittent ~10 Hz peak that scaled closely with surrounding non-tremor power bands, suggesting that the oscillatory activity was of a low signal-to-noise ratio and did not dominate or override general movement. Additionally, a gradual downward drift in oscillatory peak frequency for these whole-body tremors was observed in all subjects. The trend was adequately fit by a linear regression, with slopes ranging between –0.5 Hz/hour and –1.45 Hz/hour (Slope, 95% confidence bounds): S1: –0.0241 Hz/minute (–0.0307, –0.0175); S2: –0.0165 Hz/minute (–0.0191, –0.0139); S3: –0.008875 Hz/minute (–0.0128, –0.00497)).

In all three subjects, there was a shift in movement phenomenology from generalized movement towards a tremulous behavior with a spectral peak between 10–14 Hz, which corresponded to the whole-body tremor observed. S2 postured its recorded forearm in a flexed position; S1 and S3’s forearms were postured such that they made contact with the chair wall, but not in a way to support their body weight. This may explain the broad lower-frequency (6 Hz) oscillatory mode observed only in S2.

### Tremor-onset and tremor-magnitude across individual awake primates

**Figure 4:**
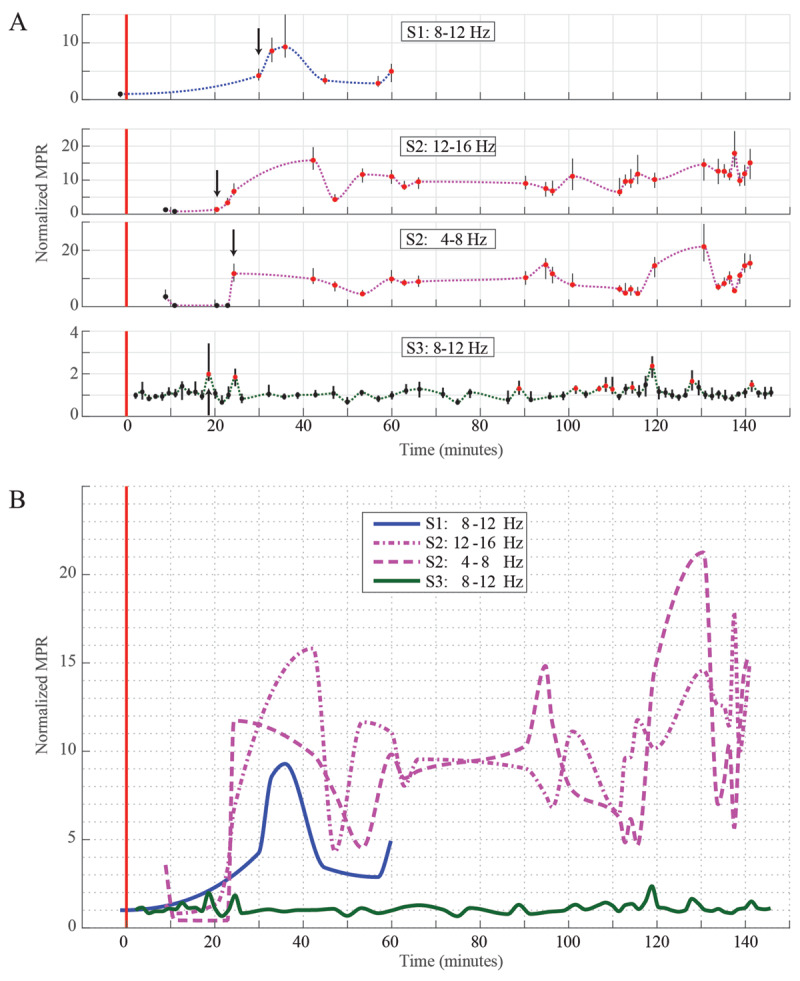
Tremor-band motion power ratio over time. Onset and magnitude dynamics of the motion power ratio (MPR) comparing a “tremor-band” of interest with 17–21 Hz content. **A:** Data points represent the median normalized MPR among all MPR samples within a segment of data (up to but not exceeding 2 minutes in duration with the time value given as the midpoint). Vertical bars indicate 95% confidence intervals. All data recorded prior to the 15 minutes post harmaline mark were treated as baseline data, from which the median is calculated for normalization and statistical-testing purposes. Red data points signify statistically signficant difference above the median baseline. Arrows indicate the earliest occurring segment of tremor-band MPR data which differed significantly from median baseline. Data points are connected with a line representing a piecewise cubic hermite interpolating polynomial (PCHIP) for visualization purposes. **B:** The aforementioned PCHIP lines from the top figure are plotted together at the same scale to emphasize the differences in magnitude for tremor-band MPR among subjects.

To identify tremor-onset times and tremor magnitude following harmaline injection, a motion power ratio (MPR) between tremor-band power and general non-oscillatory movement power (17–21 Hz) was calculated over all data segments. Inter-subject comparison was facilitated by normalizing MPR values to baseline MPR data for each subject. Tremor-onset was defined as the first time period at which the median post-injection MPR significantly differed from baseline MPR. Significant harmaline-induced tremor-onset for each subject’s power-ratio was tallied as follows: S1, 30 minutes; S2, 21 minutes (high-band) and 24 minutes (low-band); S3, 19 minutes (***Figure 4A***). S1 and S2 exhibited significant increases from baseline power-ratio that remained consistent after initial tremor-onset for the remainder of the harmaline session, whereas S3’s harmaline-induced power ratio was infrequently differentiable from baseline activity throughout the harmaline session duration. Normalized MPR in S1 and S2 remained significantly above pre-harmaline baseline at all times post-injection in a time-varying manner (***Figure 4B***).

### Harmaline dose-dependency in follow-up case study using self-paced cued reaching task

**Figure 5:**
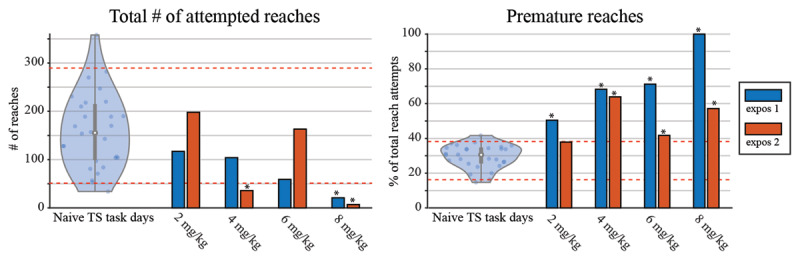
Testing self-paced cued reaching task performance under harmaline dosing conditions. Task performance deteriorated with increasing harmaline dosages compared to the performance during drug-naive sessions, with a dose-dependent trend towards fewer attempts at initiating task trials and an increasing incidence of premature reaches. Data from all drug-naive days were pooled together and displayed with 90% confidence intervals (dotted red lines), while data from harmaline-dose days are displayed separately. The central mark within each violin plot indicates the median, the edges of the vertical gray bar indicate 25th and 75th percentiles, and the entire vertical length of violine plots indicate the full range of data. * indicates harmaline-related performance data that falls outside of the 90% confidence interval of naïve performance.

During the course of harmaline sessions in subjects S1, S2, and S3, a lack of engagement for simple reaching behaviors was observed. To better understand the relationship between harmaline dosage and task performance, an 8-week dose-dependency task case study was performed in S3 (see Methods section). Increasing harmaline dosage resulted in poorer performance on the self-paced reaching task (***Figure 5***). The total number of reach-attempts within the allotted 30-minute task trended downwards as harmaline dose increased (r = –0.86, p = 0.0595, Pearson correlation between dose and average attempted reaches), getting commensurately further from the number of expected reach-attempts during the drug-naive task maintenance days that occurred between weekly harmaline sessions. Of those reach attempts observed in each session, the relative proportion of prematurely initiated reaches increased with harmaline dose (r = 0.9176, p = 0.0280, Pearson correlation between dose and average % of premature reaches). A given reach-movement towards the on-screen target was considered “premature” if the subject initiated the reach before the presentation of the on-screen target (i.e. “jumping the gun”). We note that across all dosages attempted in S3 (2–8 mg/kg), none elicited robust tremors. We further note that a period of ~18 months had elapsed between the earlier harmaline exposure study in S3 and the current behavioral-response study. These behavioral results highlight the dose-dependent non-tremor effects of harmaline on task performance in awake-behaving non-human primates.

## Discussion

In all three subjects, intramuscular harmaline injections resulted in varying levels of tremorous forelimb movements with oscillatory activity centered between 10–14 Hz with wash-in times of 20–30 minutes. However, each subject exhibited a unique response to similar doses of systemically administered harmaline, with response features characterized by differences in the amplitude, duration, and consistency of tremor. Additionally, harmaline was found to induce several non-motor features that included impairment in balance, reduced task performance, and impulsive behaviors. While harmaline has been used in several animal models, the results of this study suggest that the harmaline-primate has significant limitations as a preclinical model of Essential Tremor at intramuscular dosages that typically elicit spontaneous tremors.

### Individual heterogeneity in tremor responses to similar intramuscular doses

In this study, we observed that individual primates may have heterogeneous tremor-susceptibility to similar i.m. doses of harmaline (here 10–12 mg/kg). After administration of systemic harmaline, two of three subjects (S1, S2) exhibited consistent tremors at concentrations of at least 10–12 mg/kg, while S3 presented with a weak and intermittent tremor at 12 mg/kg. A precise explanation for this heterogeneity is not clear, though differing subject-specific tremorgenic dose thresholds are a likely factor. It is possible that higher dosages in S3 may have induced a more consistent tremorous state, but dosages above 12 mg/kg were avoided as a precaution due to observed side effects including nausea, disequilibrium, and lethargy. It is likely, though not explicitly reported, that a similar subject-specific dose-susceptibility has been encountered in prior harmaline-primate studies, prompting the practice of inducing the desired tremorous state by repeated injected boluses of i.m. harmaline until a subject’s threshold dose was reached and tremors manifested [27,28].

### Observed tremor phenotype

Though subjects were seated, tremorous behaviors observed across subjects corresponded most closely to the postural tremor phenotype, with tremors apparently distributed across all muscle groups (though accelerometry is here quantified only about the wrist). Quantitative assessment of muscle state was not performed in this study, and so the possibility of a rest-tremor (necessarily occurring in relaxed muscles) cannot be ruled out with certainty. However, tremors were observed to appear (S3) or exacerbate (S1, S2) particularly during periods when specific limbs maintained extended postures against gravity. The presence of a kinetic- or intention-tremor under the influence of harmaline was difficult to verify, as the subjects only briefly shifted seated postural positions or limb postures during recording sessions. In the case of S3 during the self-paced cued reaching task, no tremors were observed while reaching with the doses of harmaline administered.

The harmaline tremor phenotype is likely to depend on the harmaline dose delivered and the individual’s susceptibility to systemic harmaline. At relatively high doses of harmaline, subjects may transition from tremoring to tonic-clonic seizure-like behavior [36], which has been attributed to a high-dose toxicity effect. At a relatively low harmaline concentration, one study in the rat demonstrated a lack of whole-body tremor and yet the presence of localized exaggerated tremor in the forelimb during isometric contraction related to a button pushing task [21], raising the possibility of postural, intention, or task-specific tremor with low-dose harmaline. While a rest tremor was unlikely in our primates, the intravenously administered harmaline-pig has been reported to continue tremoring even after lying down, albeit at a reduced intensity [24]. Our study serves, then, as an example of the potential dependence of harmaline-tremor phenotype and tremor magnitude on harmaline dose and individual dose-susceptibility.

### Harmaline tremor frequency characteristics

Spectral analysis of tremor-accelerometry revealed the main harmaline-induced tremor observed in our primates, occurring at or above 10 Hz, was spectrally similar to that observed in other species treated with harmaline. The harmaline tremor frequency in the awake primate, as in other mammals, is therefore most comparable to the upper range of typical human ET tremor frequencies. Nevertheless, the spectral content of the primary harmaline tremor observed in the consistently tremoring S1 and S2 were similar to human ET tremors in that the oscillatory spectral power of the tremor varied little more than –/+ 2 Hz about the peak [4]. In both our primates and in the typical human ET patient, this spectrally-focused characteristic suggests a strong overriding central driver to the behavioral phenotype [37].

One unexpected result was the observation of two major oscillatory peaks in S2’s tremor accelerometry (at ~6 Hz and ~14 Hz), the higher of the two likely corresponding more closely to S1’s and S3’s harmaline-tremor. A cause for the lower ~6 Hz peak is not apparent, though we note that only this subject postured its fore limb unsupported in the air for the majority of recording time, unlike S1 and S3 whose fore-limbs often made contact with the chair.

An important refinement in comparing the harmaline primate tremor frequency characteristics to the human ET case would be the comparison of muscular activation patterns in both conditions using polyelectromyography (polyEMG). Use of polyEMG can help quantify the phasic correlations between muscle bursts among multiple antagonistic muscle pairs, allowing for comparisons with known ET activation. In non-primate animals, harmaline-tremors are known to arise from co-contraction of antagonistic muscles [23]. Both reciprocal activation and co-contraction type tremors have been observed in ET patients [38]. The muscular antagonistic activation pattern in primates during harmaline-tremor has yet to be thoroughly explored.

As a final note on tremor frequency, the peak oscillatory frequency characteristic of harmaline-tremor was found to gradually drift downwards over time for all three subjects following a roughly 30-minute wash-in period post-injection. Though these trends appeared linear within the timespan here reported, it is likely that longer durations of observation would have confirmed a gradual power-law or exponential decay in peak tremor frequency over time. A similar frequency drift was briefly reported in one other large-animal harmaline model [24], and some prior harmaline-primate studies report a minor drop in lesion-associated tremor frequency after harmaline administration [29]. There is as yet no rigorous explanation for this phenomenon, and future studies exploring experimental interventions in the harmaline-model of tremor should account for this natural drift as a confounding factor when interpreting the effects of said intervention on tremor-frequency over an hours-long timescale.

### Harmaline tremor onset, intermittency, and depression of general movement

The data showed that the initial onset of significant tremor following a 10–12 mg/kg intramuscular dose of harmaline was 20–30 minutes. These onset times were similar to those observed in harmaline rodents with intraperitoneal and subcutaneous delivery [20,21,39], but significantly longer than the onset time in other large animal models using intravenous delivery, where the onset can occur within seconds to several minutes [24].

The intensity of harmaline-tremors varied over time for both S1 and S2, while S3’s tremors were best described as weak and intermittent. Such intermittent tremors were difficult to quantify in our PSD estimates due to the averaging effect over time, but relatively noticeable tremor episodes were occasionally apparent between long spans of quiescence (***Figure 3***). It is therefore possible that harmaline-tremor tended to appear (or was exacerbated in the case of S1 and S2) during times of increased active kinetic or postural behaviors.

The intermittency-effect in harmaline tremors is well known in the rat [40]. It has been previously posited that this intermittency is due to a combination of a primarily action-tremor phenotype in harmaline tremors and a learned action-reduction behavior on the part of subjects as a way to avoid potentially aversive sensations (e.g. vertigo) during movement under systemic harmaline [41]. Consistent with this interpretation, rodents tend to exhibit decreased locomotor activity while under sufficient doses of systemic harmaline [42] as tremors begin to manifest.

This explanation for the time-varying intensity and intermittency of tremors may be consistent with the present results, considering that after administering harmaline doses in the upper range of our study (10–12 mg/kg), our subjects exhibited a marked reduction in general movement relative to baseline state. Moreover, subjects exhibited signs of nausea and behaviors suggestive of vertigo, except at the lowest dose of 2 mg/kg. The reduction in task engagement seen in the self-paced cued reach task in S3 lends credence to the notion of a harmaline-related aversion to movement. There is therefore a possibility that varying tremor intensity and intermittency among subjects was due to differing degrees of success at avoiding aversive movement-related sensations. Such an effect, if present, adds a learned-behavioral confound when interpreting inter-subject differences in tremor intensity and intermittency.

### Behavioral reach-task under harmaline: dose-dependent task engagement

Engagement with the behavioral task was found to deteriorate with increasing doses of harmaline in S3. At harmaline dosages high enough to elicit spontaneous tremors as found in the first experiment, primate engagement with a volitional task would be unreliable. The willingness or ability to engage with the task may be mediated by potential non-tremor effects of harmaline in the primate. Given that harmaline is known to accumulate non-specifically throughout the volume of brain tissue [43], harmaline may potentially elicit effects on brain regions besides the known changes in the inferior olive.

A reduced engagement with the reaching task as an avoidance behavior for harmaline dose dependent vertigo is consistent with motor-avoidance behaviors described in freely moving harmaline-rats [41], freely-moving harmaline pigs [24], and in the disturbed balance observed among our subjects. Thus a lack of motivation for the task may be due to a combination of vertigo-aversion, nausea, nystagmus, and the inability to fix gaze on a target at tremor and sub-tremor dosages of systemic harmaline [31,32,43]. Sufficient quantities of harmaline have been shown to cause altered states of consciousness in humans and other mammals [44,45]. For doses between 8–12 mg/kg, our primates exhibited a “detached” affect, with a lack of focused gaze or interest in immediate surroundings (with varying degrees of nystagmus). The lack of dose-dependent tremor intensity or duration in S3 may have stemmed from the subject requiring doses greater than those used in the study or from the subject being resistant to the tremorgenic effects of harmaline.

### Limitations

In evaluating the conclusions of this study, one must consider the relatively small cohort of primates and the limited number of doses tested. To better characterize the range of individual harmaline tremorgenic dose-susceptibility, future studies may consider a fuller dose-stepping protocol across a larger number of subjects. In addition, individual heterogeneity in harmaline-response may have been affected by factors of weight, fat to muscle ratio, and overall physical condition. Accelerometry recordings were episodic in nature rather than continuous, with variable stretches of unrecorded time, in this study. It is possible that tremorous data may have gone unrecorded. A potentially important factor of harmaline-tolerance may also have played a role in the observed tremor manifestations and task performance. Though tolerance to harmaline exposure has not been explicitly confirmed for rhesus macaque (or homo sapiens for that matter), it has been reported at least once in baboon [32], pig [24], and often in rat [20,40]. However, tolerance to repeated harmaline exposure in rat has been successfully managed in at least one study focusing on low-dose harmaline effects [21], and in baboon by spacing out harmaline administrations by one-week periods, as we did here for the behavioral task case study. Ultimately, the harmaline-tolerance effect in non-human primates would be best studied by an experimental design allowing for assessment of the factors of dose concentration, dosing intervals, and number of exposures, as well as their likely interactions.

Despite widespread use, systemic harmaline administration is as yet an imperfect model of the chronic human ET syndrome [46], and future work should seek to improve the translatability of the model to human tremor disorders. First, the pharmacological limitations of harmaline itself must be acknowledged. The motor effects of harmaline are acute in nature rather than chronic, with tremors being reversible to baseline conditions well within 24 hours of administration unlike the chronic characteristic of clinical tremor disorders. A potential tolerance to repeated harmaline exposure at spontaneous-tremor doses can also limit the utility of the drug for longitudinal or repeated-measures study designs. Importantly, spontaneous-tremor doses of the drug cause behavioral changes that can hamper the study of tremor characteristics in subjects performing specific tasks similar to those that elicit tremor in ET patients. Second, the phenomenology of the tremor itself is highly dependent on drug dose, time course, and individual subject susceptibility. The intensity of harmaline-tremors (but not the frequency) has been reported to be dose-dependent [24], with tremor features also depending on both the time elapsed since injection and the individual awake-behavioral responses of subjects. Furthermore, the tremors induced by most harmaline dosing approaches in animal models are distributed whole-body tremors rather than the limb-localized postural and kinetic tremors typical of human ET.

All the above notwithstanding, our observations in this study serve as a helpful waypoint in the further development of a pharmacological primate model of tremor. The generation of strong spontaneous tremors with harmaline and related alkaloids has also been observed in human subjects [8,47], with systemic administration often causing dysphoria and hallucinations, or bradycardia and hypotension often concomitant with a sedative effect [47,48]. The dose-dependent lack of task engagement seen in this study could be related to both the psychological and physiological alterations seen in human subjects, and similar depression of activity has been observed in other large mammals at high intravenous doses of harmaline [24]. A low-dose harmaline study in the rat [21] suggests that specific tremor-eliciting behaviors may be repeatedly reproduced in the same subject consistently. Further studies in primate are warranted, perhaps utilizing such a low-dose paradigm to allow for compliance in behavioral tasks and a repeated-measures design using harmaline or other indole-alkaloids [47].
